# Novel lissencephaly-associated *NDEL1* variant reveals distinct roles of NDE1 and NDEL1 in nucleokinesis and human cortical malformations

**DOI:** 10.1007/s00401-023-02665-y

**Published:** 2024-01-09

**Authors:** Meng-Han Tsai, Hao-Chen Ke, Wan-Cian Lin, Fang-Shin Nian, Chia-Wei Huang, Haw-Yuan Cheng, Chi-Sin Hsu, Tiziana Granata, Chien-Hui Chang, Barbara Castellotti, Shin-Yi Lin, Fabio M. Doniselli, Cheng-Ju Lu, Silvana Franceschetti, Francesca Ragona, Pei-Shan Hou, Laura Canafoglia, Chien-Yi Tung, Mei-Hsuan Lee, Won-Jing Wang, Jin-Wu Tsai

**Affiliations:** 1 Department of Neurology, Kaohsiung Chang Gung Memorial Hospital, Kaohsiung, Taiwan; 2 School of Medicine, College of Medicine, Chang Gung University, Taoyuan, Taiwan; 3 Institute of Brain Science, College of Medicine, National Yang Ming Chiao Tung University, Taipei, Taiwan; 4 Department of Medical Education, Linkou Chang Gung Memorial Hospital, Taoyuan, Taiwan; 5 Faculty of Medicine, College of Medicine, National Yang Ming Chiao Tung University, Taipei, Taiwan; 6 Institute of Clinical Medicine, College of Medicine, National Yang Ming Chiao Tung University, Taipei, Taiwan; 7 Advanced Therapeutics Research Center, National Yang Ming Chiao Tung University, Taipei, Taiwan; 8 Genomics Center for Clinical and Biotechnological Applications, Cancer Progression Research Center, National Yang Ming Chiao Tung University, Taipei, Taiwan; 9 Department of Paediatric Neuroscience, European Reference Network EPIcare, Fondazione IRCCS Istituto Neurologico Carlo Besta, Milan, Italy; 10 Unit of Medical Genetics and Neurogenetics, Fondazione IRCCS Istituto Neurologico Carlo Besta, Milan, Italy; 11 Department of Biotechnology and Laboratory Science in Medicine, National Yang Ming Chiao Tung University, Taipei, Taiwan; 12 Neuroradiology Unit, Fondazione IRCCS Istituto Neurologico Carlo Besta, Milan, Italy; 13 Integrated Diagnostics for Epilepsy, Department of Diagnostic and Technology, European Reference Network EPIcare, Fondazione IRCCS Istituto Neurologico Carlo Besta, Milan, Italy; 14 Institute of Anatomy and Cell Biology, College of Medicine, National Yang Ming Chiao Tung University, Taipei, Taiwan; 15 Brain Research Center, National Yang Ming Chiao Tung University, Taipei, Taiwan; 16 Institute of Biochemistry and Molecule Biology, College of Life Science, National Yang Ming Chiao Tung University, Taipei, Taiwan; 17 Department of Biological Science and Technology, College of Biological Science and Technology, National Yang Ming Chiao Tung University, Hsinchu, Taiwan

**Keywords:** Lissencephaly, Microcephaly, Microlissencephaly, NDEL1, NDE1, LIS1, PAFAH1B1, Dynein, DYNC1H1, Neuronal migration, Nucleokinesis, Interkinetic nuclear migration, Radial glial cell

## Abstract

The development of the cerebral cortex involves a series of dynamic events, including cell proliferation and migration, which rely on the motor protein dynein and its regulators NDE1 and NDEL1. While the loss of function in NDE1 leads to microcephaly-related malformations of cortical development (MCDs), *NDEL1* variants have not been detected in MCD patients. Here, we identified two patients with pachygyria, with or without subcortical band heterotopia (SBH), carrying the same de novo somatic mosaic *NDEL1* variant, p.Arg105Pro (p.R105P). Through single-cell RNA sequencing and spatial transcriptomic analysis, we observed complementary expression of *Nde1*/*NDE1* and *Ndel1*/*NDEL1* in neural progenitors and post-mitotic neurons, respectively. *Ndel1* knockdown by in utero electroporation resulted in impaired neuronal migration, a phenotype that could not be rescued by p.R105P. Remarkably, p.R105P expression alone strongly disrupted neuronal migration, increased the length of the leading process, and impaired nucleus–centrosome coupling, suggesting a failure in nucleokinesis. Mechanistically, p.R105P disrupted NDEL1 binding to the dynein regulator LIS1. This study identifies the first lissencephaly-associated *NDEL1* variant and sheds light on the distinct roles of NDE1 and NDEL1 in nucleokinesis and MCD pathogenesis.

**Supplementary Information:**

The online version contains supplementary material available at 10.1007/s00401-023-02665-y.

## Introduction

One of the most distinctive features of the human brain is the multilayered organization of the cerebral cortex and the complex folding of its outer surface. The mammalian cerebral cortex comprises six layers, each containing functionally distinct pyramidal neurons. The development of the cerebral cortex involves a series of highly regulated steps, including neural progenitor proliferation, differentiation, and neuronal migration. During the massive expansion of the cerebral cortex, radial glial cells (RGCs) proliferate in the ventricular zone (VZ) of the telencephalon, first symmetrically to expand the progenitor pool and later asymmetrically to produce post-mitotic neurons, intermediate progenitors (IPs), or other progenitors [38, 63, 74]. As RGCs proliferate, their nucleus oscillates in the VZ, a process termed interkinetic nuclear migration (INM), during the cell cycle. In the G1 phase, the nucleus of RGCs migrates from the ventricular surface to the subventricular zone (SVZ) to undergo the S phase. In the G2 phase, the nucleus moves inward to the ventricular surface, where cell division occurs. In asymmetric divisions that give rise to neurons, these post-mitotic neurons then migrate outward along the radial fibers of the RGCs to populate the cerebral cortex.

Migrating neurons in the developing cerebral cortex exhibit a unique migratory cycle termed “two-stroke movement” [58, 66]. Neuronal precursors first extend a leading process toward the cortical plate (CP) [31]; the centrosome then moves into an enlarged swelling of the leading process. Subsequently, the nucleus moves into the leading process, a phenomenon known as “nucleokinesis” [6, 53, 58, 66]. During nucleokinesis, the nucleus moves along trailing microtubules that originate from the centrosome and form a cage-like structure surrounding the nucleus. The coupling of centrosomal and nuclear movements is essential for directed neuronal migration [6, 68]. Cytoplasmic dynein, regulated by associated proteins such as LIS1, NDE1, and its paralog NDEL1, generates forces along microtubules and pulls the nucleus toward the centrosome [64, 66].

Mutations in genes encoding cytoplasmic dynein (*DYNC1H1*) and its adaptors have been associated with various malformations of cortical development (MCDs) in humans. Heterozygous deletions or mutations of *LIS1* (also known as *PAFAH1B1*) result in lissencephaly (smooth brain) [2, 5, 18, 24, 46, 47, 61]; biallelic mutations or deletions in *NDE1* have been shown to result in microcephaly (small brain) [46], microlissencephaly [2, 5] or microhydranencephaly [24]. To date, no *NDEL1* variants have been found in human patients with cortical malformations. Interestingly, *Nde1* knockout mice show a reduction in cortical thickness [21], whereas *Ndel1* knockout mice are embryonically lethal [51]. Therefore, it has been suggested that complete loss of NDEL1 may not be viable in humans [51]. However, the differences between NDE1 and NDEL1 in modulating cortical development and brain malformation in humans remain unclear.

Through whole-exome sequencing (WES), this study identified, for the first time, two unrelated patients with pachygyria, with or without subcortical band heterotopia (SBH, also known as double cortex), both carrying the same de novo* NDEL1* (NM_030808.5) missense variant c.314G > C; p.Arg105Pro (p.R105P). To elucidate why *NDE1* and *NDEL1* variants cause different human phenotypes, we examined their expression patterns in the developing cerebral cortex using spatial transcriptomic analysis and single-cell RNA sequencing (scRNA-seq). Surprisingly, we found that *Nde1*/*NDE1* was specifically expressed in RGCs within the VZ in a cell-cycle-dependent manner, whereas *Ndel1*/*NDEL1* was found in post-mitotic neurons within the IZ and CP. We also demonstrated that expression of the *NDEL1* p.R105P variant disrupted neuronal migration and the interaction of NDEL1 with the dynein regulator LIS1, which in turn may affect the normal functions of the cytoplasmic dynein complex. These results resolve a long-standing puzzle of the distinct roles of NDE1 and NDEL1 in cortical development and provide the cellular and molecular mechanisms of how pathogenic *NDEL1* variants cause neuronal migration defects and human lissencephaly.

## Material and methods

### Ethics and patient recruitment

The cases were referred to the genetic study program at the Department of Neurology at Kaohsiung Chang Gung Memorial Hospital, Taiwan, and the Fondazione IRCCS Istituto Neurologico Carlo Besta, Milan, Italy. Clinical phenotyping was performed by direct interviews and reviews of medical records. Neuroimaging studies were performed as part of the clinical routine. The study was approved by the local human research ethics committees, and written consent was obtained from the parents.

### Whole-exome sequencing and Sanger validation

Whole-exome sequencing was performed on Case 1 as previously described [69–71]. In short, genome DNA was captured using IDT xGen Exome Research Panel V2 and sequenced using the Illumina NovaSeq 6000 platform. Reads were mapped to the human reference genome (hg19), followed by a standardized bioinformatics pipeline [70]. Variants were called using FreeBayes, annotated using ANNOVAR [73], and filtered using the *Exome Aggregation Consortium (ExAc) and the Genome Aggregation Database (gnomAD). Filtered ultra-rare variants were prioritized using two phenotype-based bioinformatic tools: Phenolyzer and VarElect [60, 77]. Potential variants were validated using Sanger sequencing using an ABI 3730XL DNA analyzer. WES on Case 2 was performed through the Epi25 Collaborative.

### Single-cell RNA sequencing (scRNA-seq) and analysis

C57BL/6 embryonic mouse brains at E13.5 and E15.5 were used for scRNA-seq experiments. The sample preparation procedure was conducted following the handbook of the Neural Tissue Dissociation Kit (130–092-628, Myltenyi Biotec). Briefly, brain tissue was dissociated into single cells by gentleMACS™ Octo Dissociator with Heaters. The library for scRNA-seq was constructed by Chromium Single Cell 3’ Library kit (10 × Genomics Chromium Single Cell 3’ Reagent Kit V.3.1, no. 1000121) following the manufacturer’s recommendations. Then reverse-transcribed complementary DNA was sequenced using Illumina NovaSeq 6000 (Illumina). The quality control and data processing were performed using the 10 × Genomics Cell Ranger pipeline package. mRNA from 8879 cells from the E13.5 brain was sequenced with a total of 149,175 reads and a median of 3355 genes per cell. For the E15.5 brain, 10,319 cells were sequenced with a total of 109,277 reads and a median of 3943 genes per cell. For human gene profiling, we used scRNA-seq data obtained from the future somatosensory area of the human embryonic forebrain at GW18 [7].

The dataset of E13.5 and E15.5 mouse embryonic cortex and GW18 human embryonic cortex was analyzed using Seurat v4.0 R toolkit with RStudio software. The number of principal components was calculated by the ElbowPlot function. Thirty, thirty-five, and nineteen principal components were used to generate the E13.5, E15.5, and human GW18 UMAPs, respectively. Three developmental states, i.e., progenitors, transient stage and post-mitotic neurons, were designated based on marker genes for progenitors (Pax6/PAX6 and Fabp7/FABP7), transient stage (Eomes/EOMES), and post-mitotic cells (Neurod6/NEUROD6), as well as cortical genes (Tbr1/TBR1, Bcl11b/BCL11B, and Satb2/SATB2). Gene expression data and violin plots were calculated and visualized using the Seurat v4.0 and Nebulosa R toolkit [3].

### Spatial transcriptome analysis

Spatial transcriptome analysis (Visium, 10 × Genomics) was performed on the brain sample of an embryonic C57BL/6 mouse at E15.5. The tissue processing procedure was carried out according to the manufacturer’s instructions. Briefly, the brain tissue was freshly taken out and embedded immediately in the optimal cutting temperature (OCT) compound, followed by freezing in isopentane (Sigma-Aldrich) bath in liquid nitrogen. 10-μm-thick brain sections were obtained using a cryostat (NX70; ThermoFisher) in a sagittal plane and placed on pre-chilled Visium spatial tissue optimization (TO) slides or gene expression (GE) slides (10X Genomics). Images of histology HE staining were obtained by inverted microscope IX83 (Olympus, 10x). Sequencing of cDNA libraries was performed using a NovaSeq 6000 System (Illumina) and the NovaSeq S4 Reagent Kit (300 cycles). In our experiment, the number of spots covered by the tissue was 1189 out of 5000 (23.8%), the average number of reads was 191,168, and the medium number of genes was 3092. The initial processing of Visium data, including raw FASTQ files and images, was carried out using Space Ranger software (Version 1.2.1), and gene alignment was conducted using the mouse reference genome (MM10). The following data were processed and analyzed by Loupe Browser 6.4.0.

### Constructs and mutagenesis

To knock down the expression level of *Ndel1* in mouse embryos, five shRNA targeting mouse *Ndel1* were used as knockdown constructs, and a non-targeting sequence was used as the control for in utero electroporation experiment. All constructs were purchased from Academia Sinica RNAi core. Target sequences of *Ndel1* shRNA were as follows: shCtrl: CCTAAGGTTAAGTCGCCCTCG, sh*Ndel1*#13 (CDS): GCGAGCAACTTTCTTCCATAA, sh*Ndel1*#14 (CDS): GCATCCCGCAAATCTTATGTT, sh*Ndel1*#16 (3’ UTR): TTGCTGTGCTGATAGGATTTA, sh*Ndel1*#69 (3’ UTR): GCGATATCAATACTGGCTATT, sh*Ndel1*#85 (CDS): GTATGAAGTGGAGGCGTTAAA. To overexpress NDEL1 in HEK293T cells, full-length mouse *Ndel1* WT cDNA (RefSeq: NM_023668.3) was first cloned into pEGFP-C1 (Clontech Laboratories, Inc.) and become pEGFP-NDEL1. To generate the cDNA clones of *Ndel1* variants (p.R105P, p.R105Q, and p.R105W), the mutagenesis PCR protocol was conducted by following the instructions from the QuikChange II Site-Directed Mutagenesis Kit (Agilent Technologies). Constructs expressed in cells are as follows: pEGFP-NDEL1, pEGFP-R105P, pEGFP-R105Q, and pEGFP-R105W. Primer sequences for *Ndel1* p.R105P, p.R105Q, and p.R105W mutagenesis (5’ to 3’) are as follows: p.R105P forward: ATTTAAGTCAGACCCCGGCCATTAAGGAGC, p.R105P reverse: GCTCCTTAATGGCCGGGGTCTGACTTAAAT, p.R105Q forward: TGATTTAAGTCAGACCCAGGCCATTAAGGAGCAAC, p.R105Q reverse: GTTGCTCCTTAATGGCCTGGGTCTGACTTAAATCA, p.R105W forward: GATGATTTAAGTCAGACCTGGGCCATTAAGGAGCAAC, p.R105W reverse: GTTGCTCCTTAATGGCCCAGGTCTGACTTAAATCATC.

For expression of *Ndel1* in mouse embryos, *Ndel1* WT and variants were subcloned from the pEGFP-C1 backbone into pCAGIG (Addgene #11,159) or pNeuroD-ires-GFP (Addgene #61,403). Constructs expressed in mouse embryos are as follows: pCAGIG-NDEL1, pCAGIG-R105P, pCAGIG-R105Q, pCAGIG-R105W, pNeuroD-NDEL1, and pNeuroD-R105P.

### In utero electroporation

In utero electroporation was performed according to previous reports [13, 28, 37, 67, 70, 71]. Briefly, timed pregnant ICR mice at E14.5 were anesthetized by isoflurane, and the skin, as well as muscular tissue of the abdomen, was further incised to expose the uterine of mice. Plasmid DNA encoding cDNA (0.7 μg/μl) or shRNA (1.5 μg/μl) was then injected into one of the lateral ventricles of the mouse embryos through a glass micropipette. Forceps-type electrodes were used to induce five 50 ms pulses with an interval of 450 ms at a voltage of 50 V on the embryonic mouse brains. After electroporation, the uterine horns were placed back into the abdominal cavity carefully, and the incision was closed by suture. The embryos could develop normally in the uterus and were given birth by the mother occurred naturally. The brains of the electroporated mice were harvested at E18.5 or P7. The protocol is approved by the Institutional Animal Care and Use Committee (IACUC) of National Yang Ming Chiao Tung University (No. 1080626).

### Cell culture and transfection

HEK293T and U2OS cells were cultured in DMEM (Life Technologies, USA) supplemented with 10% FBS at 37℃ in humidified air containing 5% CO_2_ [36, 72]. To transfect pEGFP-C1 constructs into cell culture, cells were placed in 6 cm Petri dishes with a density of 70–80%. The Lipofectamine 3000 lipofection reagent (Life Technologies, USA) was used, and cells were harvested 24 h after transfection. To test the knockdown efficiency of shRNA constructs, dissociated neuronal cultures were collected from the cerebral cortex of embryonic mice. The primary culture neurons were infected by lentivirus packaged with shNdel1 at DIV 2 and removed after 24 h by replacing fresh medium. Proteins from primary cultures were extracted at DIV 6 based on a previous report [44].

### Protein extraction and western blot

After transfection, the cells were washed with PBS and were lysed with RIPA lysis buffer, which contains 10% protease inhibitor and 10% phosphatase inhibitor. The collected protein lysate was then sonicated for 30 min and rotated on a 4℃ rotor for 1 h. To further remove impurities, the protein lysate was centrifuged at 4℃, 15,000 g for 15 min, and the supernatant was extracted.

A BCA assay kit (Thermo Fisher Scientific, USA) was used for the quantification of the protein samples. 30 μg of protein samples mixed with 6 × loading buffer were loaded and separated by electrophoresis, which was carried out in 12% gel SDS-PAGE and transferred onto a PVDF membrane (Immobilon). The transferred membrane was soaked in 0.1% TBS-T containing 5% skim milk and 3% BSA for blocking and incubated in primary antibodies overnight at 4 °C. After washing the transferred membrane three times, rabbit and mouse horseradish peroxidase (HRP) conjugated secondary antibodies were incubated for 2 h at room temperature to amplify the signal. The antibodies used and their concentrations were as follows: NDEL1(Rabbit polyclonal, Abcam, ab25959) 1:1000, NDE1 (Rabbit polyclonal, Proteintech, 10,233–1-AP) 1:1000, LIS1 (Goat polyclonal, Sigma-Aldrich, SAB2500597) 1:1000, Beta-actin (Mouse monoclonal, Proteintech, 66,009–1-Ig) 1:10,000, and Alpha tubulin (Mouse monoclonal, Proteintech, 66,031–1-Ig) 1:10,000.

### Co-immunoprecipitation

HEK293T cells were lysed using the lysis buffer containing 10% protease inhibitors and phosphatase inhibitors, then incubated at 4 °C for 1 h, followed by centrifugation at 20,000 g speed for 15 min. After centrifugation, the supernatant was collected for further analysis. GFP Trap system was used for co-immunoprecipitation; protein concentration was 1 μg/μl in every sample to give a total quantity of 400 μl of protein in the lysis buffer for each. A mixture of protein samples and washed GFP beads were placed on a rotator platform at 4 °C for 15–18 h. After incubation, the protein and the beads were separated by a magnetic stand, and the beads were washed with wash buffer six times to reduce noise. All procedures were conducted on ice or in a cold room. Last, samples were eluted from the beads by adding 1X sample dye and boiled in 100 °C dry baths for 10 min.

### Immunofluorescence staining

Before brain slice staining, mouse embryos were harvested and perfused through the left cardiac ventricle with 4% PFA in PBS. Mouse brains were further fixed in 4% PFA in PBS at 4 °C overnight. Brain slices were sectioned at 100 μm in thickness on a Vibratome (Leica) and stored in PBS with 0.05% NaN_3_ (Sigma) at 4 °C. For immunofluorescence staining, selected brain slices were washed in PBS and then soaked in 0.2% PBS-T for 30 min. For blocking, sections were incubated in 10% NGS and 5% BSA in PBS for 1 h at room temperature, followed by immersion in primary antibodies for 2 days at 4 °C. Sections were washed in 0.2% Triton X-100 in PBS and incubated with the secondary antibody for 2 h at room temperature. DNA was stained with 4',6- diamidino-2-phenylindole (DAPI) (Molecular Probes). The antibodies and concentration used were: 1) primary antibodies: NeuN (Rabbit monoclonal, Sigma-Aldrich, ZRB377) 1:500; gamma-tubulin (Rabbit polyclonal, Abcam, ab11317) 1:500; Brn2 (Mouse monoclonal, Santa Cruz, sc-393324) 1:500; and 2) secondary antibodies: Anti-Rabbit IgG, Alexa Fluor 546 (Goat, Invitrogen, A-11035) 1:1000; Anti-Mouse IgG, Alexa Fluor 546 (Goat, Invitrogen, A-11030) 1:1000.

### Microscopy and image analysis

All images were taken by confocal microscopy (ZEISS LSM 700), with a 20X Plan-Apochromat NA = 0.8 objective. The DAPI signal was excited by a 405 nm Diode laser; the green fluorescence signal (GFP) was excited by a 488 nm Argon laser; and the red fluorescence signal (Cent2-DsRed or Alexa 546) was excited by a 555 nm HeNe laser. The distribution of the electroporated neurons was analyzed with ZEN (Zeiss) and ImageJ software.

### Statistical analysis

All the statistical data were managed through the software GraphPad Prism 8.0. Student *t *test was used to compare the differences between two experimental conditions, while one-way ANOVA was used to compare the differences among more than two groups. Statistical significance was set as *p* < 0.05.

## Results

### De novo postzygotic mosaic variant in *NDEL1* identified in patients with pachygyria, with or without SBH

**Fig. 1:**
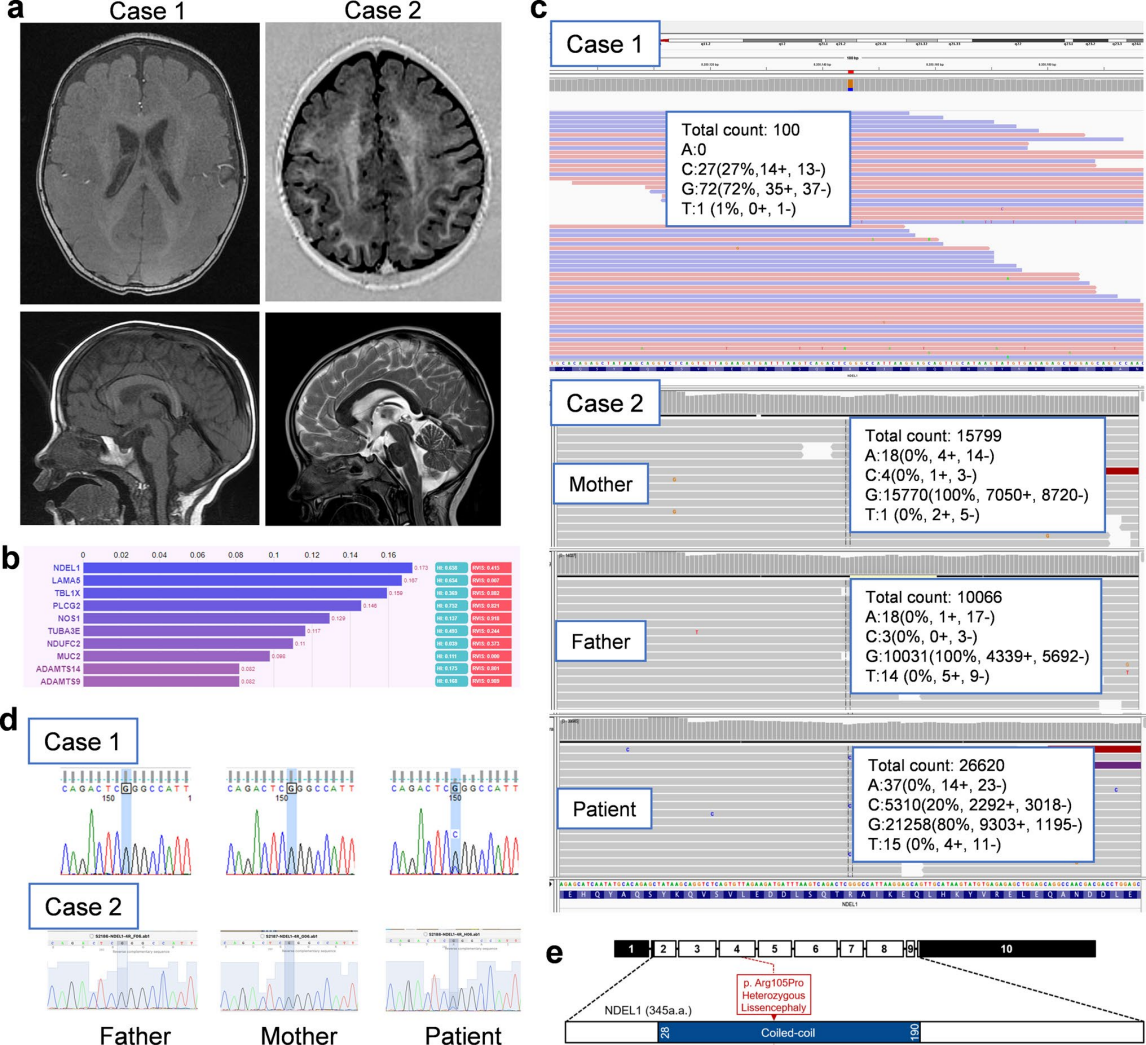
Identification of a de novo pathogenic *NDEL1* variant in patients with pachygyria and subcortical heterotopia. **a** Brain MRI of the patients. In Case 1, the axial T1 image at 9 months of age shows diffuse, thick (> 10 mm) pachygyria, with the posterior regions more affected than the anterior regions. Sagittal view at 1 year and 8 months of age) shows a mildly thickened corpus callosum. The basal ganglion, brain stem, and cerebellum show no dysplasia or hypoplasia. In Case 2, the axial T1 imaging at 3 years old shows anterior pachygyria and posterior subcortical band heterotopia (double cortex). The sagittal T2 image shows a mildly thickened corpus callosum. **b** The bar graph shows the top ten genes prioritized by the Phenolyzer tool, ranking *NDEL1* as the most important gene associated with lissencephaly. The X-axis represents the weighted score calculated by the Phenolyzer tool (https://phenolyzer.wglab.org) [77]. HI: Haploinsufficiency Score; RVIS: Residual Variant Intolerance Score. *NDEL1* was also ranked as the most important gene by the VarElect tool (data not shown). **c** The WES study identified the same de novo missense variant in *NDEL1*: exon4: c.314G > C, p.R105P, in both patients with different mosaicism levels (25–30% in Case and 20–25% in Case 2). **d** Sanger sequencing of the patients and their parents confirms that the missense variant was de novo in origin. **e** Schematic representation of the relative location of p.R105P to known protein domains in NDEL1

Case 1: A 5-year-old boy presented with infantile hypotonia, developmental delay evident at 4 months of age, and epilepsy onset at 6 months of age. He had multiple seizure types, including infantile spasms, absence seizures, myoclonic seizures, and atonic seizures that were resistant to multiple anti-seizure medications (ASMs). He exhibited no language development and began walking under orthosis assistance at 5 years old. Head circumference fell within the normal range (40–50 percentile) at different ages. There was no family history of epilepsy or intellectual disability. Brain magnetic resonance imaging (MRI), acquired at 20 months of age, showed diffuse thick pachygyria, with the posterior brain region more severely affected than the anterior. The corpus callosum showed mild thickening (more than 2 standard deviations, measured at the level of the genu, body, isthmus, and splenium) [22]. The brain stem, cerebellum, and basal ganglion appeared relatively normal **(**Fig. 1a**)**.

Case 2: A 9-year-old girl, born after an uneventful pregnancy, had an uneventful perinatal period and no family history of neurological disorders. She exhibited mild psychomotor and language delays from the first year of life. Epilepsy onset occurred at 28 months, initially with focal seizures, and later, she developed multiple seizure types, including tonic–clonic seizures and clusters of spasms. These seizures were resistant to multiple ASMs. She presented with moderate intellectual disability and severe language impairment and was capable of producing only single words. She had a normal neurological examination, and her head circumference fell within the 25th percentile, similar to other growth parameters. She was able to walk independently, albeit with mild clumsiness. Brain MRI, acquired at 3 years of age, showed anterior pachygyria and posterior double cortex. The corpus callosum was abnormally thick, measuring over 2 standard deviations [22]. The brain stem, cerebellum, and basal ganglion appeared relatively normal **(**Fig. 1a**)**.

WES was initially performed on Case 1 and identified 109 coding or splicing ultra-rare variants in 103 genes that were not present in either the exome aggregation database (ExAc) or the genome aggregation database (gnomAD) and were absent in 160 control exomes that were sequenced and analyzed locally using the same methods. None of these variants were known to cause lissencephaly. Two methods were used to prioritize variants associated with lissencephaly: VarElect and Phenolyzer [60, 77]. Both tools placed the nuclear distribution element-like 1 (*NDEL1*) gene (RefSeq: NM_030808) at the top of the list of candidate genes **(**Fig. 1b**)**. The novel somatic mosaic (25–30%) missense variant (exon4: c.314G > C, p.R105P) **(**Fig. 1c**)** was confirmed as de novo in the patient and his parents by Sanger sequencing **(**Fig. 1d**)**. The variant was predicted to be deleterious/damaging/disease-causing by PROVEAN, Polyphen2, and MutationTaster2 with a CADD score of 32. This variant is located in the coil-coiled domain responsible for self-association and binding to LIS1 **(**Fig. 1e**)**.

Subsequently, through the Epi25 Collaborative (http://epi-25.org), we found that the same variant was present in Case 2 with 15–20% somatic mosaicism (80–85% wild-type), while it was absent in the parents. It is worth noting that the p.R105 residue has two additional missense variants present in 14 individuals within the gnomAD database, resulting in changes from arginine to tryptophan or glutamine (assessed in September 2023). Although *NDEL1* variants have never been reported to cause lissencephaly in humans, previous studies have strongly linked *NDEL1* to other lissencephaly genes such as *LIS1* [17, 27, 81] and *DYNC1H1* [32, 51], making *NDEL1* a good candidate gene for lissencephaly.

### Complementary expression of *NDE1* and *NDEL1* in the developing cerebral cortex

**Fig. 2:**
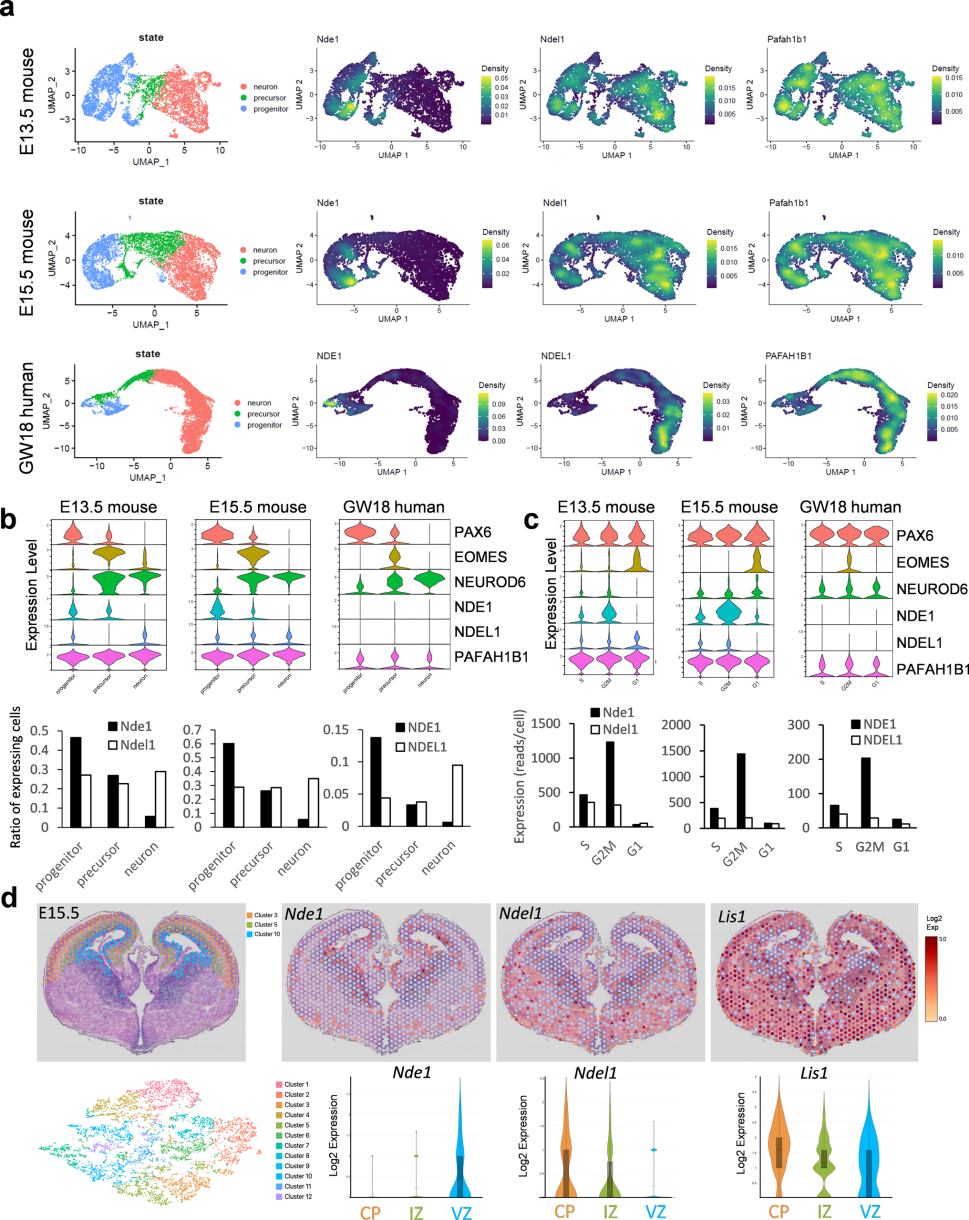
Expression of Nde1/NDE1 and Ndel1/NDEL1 in the developing mouse and human cortices. **a** UMAP plot of scRNA-seq of mouse cortex at E13.5 and E15.5 and human cortex at GW18. Three developmental states (progenitors, precursors, and neurons) are designated based on their marker genes (Supplementary Fig. 1). Heat maps of *Nde1*/*NDE1*, *Ndel1*/*NDEL1*, and *Pafah1b1*/*PAFAH1B1* (*Lis1*/*LIS1*) expression show specific expression patterns. **b** Violin plots of *Nde1*/*NDE1*, *Ndel1*/*NDEL1*, and *Pafah1b1*/*PAFAH1B1* (*Lis1*/*LIS1*) expression at three neuronal developmental states (top). The markers *Pax6*/*PAX6*, *Eomes*/*EOMES*, and *Neurod6*/*NEUROD6* are shown to distinguish these developmental stages. Bar graphs show the ratio of cells expressing *Nde1*/*NDE1* and *Ndel1*/*NDEL1* at each developmental state (bottom). Both plots show preferential expression of *Nde1*/*NDE1* in the progenitor state. **c** Violin plots of *Nde1*/*NDE1*, *Ndel1*/*NDEL1*, and *Pafah1b1*/*PAFAH1B1* (*Lis1*/*LIS1*) expression in progenitors at different cell cycle stages (top). Bar graphs show the expression of *Nde1*/*NDE1* and *Ndel1*/*NDEL1* at each cell cycle stage (bottom). Both plots show relatively high expression of *Nde1*/*NDE1* at the G2/M phase. **d** Gene expression profiles in mouse telencephalon at E15.5. Left: Spatial distribution and UMAP plot of individual capture dots grouped into 12 clusters based on their expression profiles. Right: The spatial distribution and violin plot of the expression of *Nde1*, *Ndel1*, and *Lis1* in the dorsal telencephalon. While *Nde1* is strongly expressed in the VZ, *Ndel1* is mainly expressed in the IZ and CP. The color scale represents the log-normalization of the unique molecular identifier (UMI) count (also see Supplementary Fig. 2)

To explore the possible reasons for the different phenotypes of *NDE1* and *NDEL1* mutations in humans, we first examined the expression patterns of *Nde1*/*NDE1* and *Ndel1*/*NDEL1* in the developing mouse and human cortices at single-cell resolution using scRNA-seq [25]. For scRNA-seq in mice, single cells were isolated from the dorsal pallium at embryonic day (E)13.5 and E15.5, and their gene expression profiles were grouped into clusters **(**Fig. 2a**; Supplementary Fig. 1)**. For human gene profiling, we analyzed scRNA-seq data obtained from human embryonic forebrain at gestational week (GW) 18 [7]. To verify the cell types of each cluster, we examined the expression of neural cell markers, including *Foxg1*/*FOXG1*, *Pax6*/*PAX6*, *Emx1*/*EMX1*, *Eomes*/*EOMES*, *Neurod6*/*NEUROD6*, *Tbr1*/*TBR1*, *Bcl11b*/*BCL11B*, *Satb2*/*SATB2*, and *Fabp7*/*FABP7* **(Supplementary Fig. 1)**. Based on their expression profiles, the cells could be classified into three main types: progenitor (i.e., RGC), precursor (i.e., IP), and neuron **(**Fig. 2a**)**. We found that *Nde1* was predominantly expressed in progenitors and precursors both at E13.5 and E15.5, whereas *Ndel1* was present in all three cell types, with slightly higher expression in neurons **(**Fig. 2a, b**)**. In comparison, another lissencephaly gene, *Lis1* (*Pafah1b1*), was expressed at similar levels in all three cell types. Consistently, in the GW18 human fetus, expression of *NDE1* was enriched in progenitors, whereas *NDEL1* was more enriched in neurons (Fig. 2a, b), although the *NDE1*- and *NDEL1*-expressing cell populations in human embryonic neocortex appeared to be smaller than those in mice (Fig. 2b), possibly due to the very high neuron number in human neocortex at this stage.

Because RGCs undergo INM, which requires specific regulation of dynein motor activity, we next examined *NDE1* expression in the progenitor population at different cycle stages (Fig. 2c). We found that *Nde1* was specifically expressed at the G2/M phase in progenitors at both E13.5 and E15.5. Similarly, *NDE1* expression was strongest at the G2/M phase in the human brain at GW18. These results suggested a specific cell-cycle-dependent role of NDE1 in neural progenitor cell proliferation (Fig. 2c).

We next used spatial transcriptomic analysis, which allows whole-genome expression profiling in tissues with spatial information [12]. Four coronal brain sections from E15.5 mice were placed on a specialized RNA capture slide, on which mRNA was captured and tagged with spatial information at a hexagonal 55-μm resolution. The mRNA was then sequenced, and based on the gene expression profiles of each capture region, these capture regions could be grouped into 12 clusters **(**Fig. 2d**)**. Among the resulting 12 clusters, clusters 3, 5, and 10 corresponded nicely to the CP, IZ, and VZ of the developing cortex, respectively. Remarkably, we found that the expression of *Nde1* was largely restricted to the VZ of the developing mouse cortex. In contrast, the expression of *Ndel1* was high in the CP and IZ but lower in the VZ **(**Fig. 2d**, Supplementary Fig. 2)**. By comparison, *Lis1* was strongly expressed in all three zones. The different expression patterns of *Nde1*/*NDE1* and *Ndel1*/*NDEL1* in the developing cortex suggested their distinct roles in cortical development.

### Knockdown of *Ndel1* impaired neuronal migration during cortical development

**Fig. 3:**
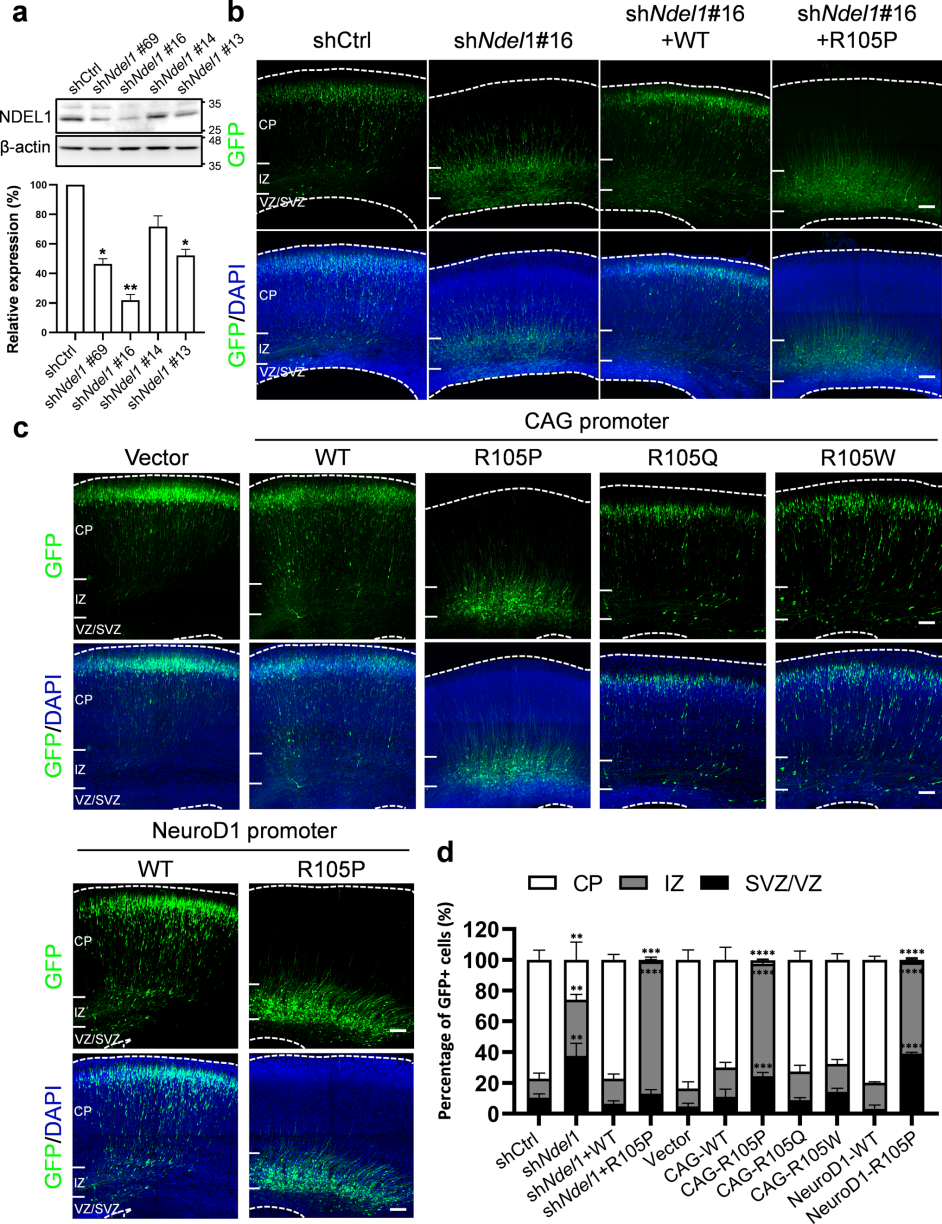
Effects of Ndel1 knockdown and p.R105P variant on neuronal distribution in embryonic mouse brains. **a** Western blot of NDEL1 protein in primary cultures at DIV 6 after infection with viruses encoding control or 4 different *Ndel1* shRNA. Beta-actin was used as the loading control. The bar graph shows relative NDEL1 expression in these cells. Error bars represent SEM. One-way ANOVA; post hoc: Bonferroni test. *: *p* < 0.05, **: *p* < 0.01. **b** Coronal sections of E18.5 brains electroporated with shCtrl or sh*Ndel1* along with GFP (green) at E14.5. While most GFP + cells reached the CP, cells expressing sh*Ndel1*#16 were mainly restricted to the IZ and VZ/SVZ. This effect was rescued by the expression of *Ndel1* WT but not the p.R105P variant. **c** Coronal sections of E18.5 brains electroporated with *Ndel1* WT, p.R105P, p.R105Q, p.R105W, or the empty vector along with GFP (green) at E14.5. GFP + cells electroporated with pCAGIG-NDEL1, pCAGIG-R105Q, pCAGIG-R105W, pNeuroD1-NDEL1(WT), or vector were mainly redistributed to the CP. However, the expression of CAG / NeuroD1 promoter-driven p.R105P caused a nearly complete blockade of neurons from migrating to the CP. Slices were stained with DAPI (blue) to mark the nuclei of the cells. Bars = 100 μm. **d** The bar graph shows the percentage of GFP + cells in the CP, IZ, and VZ 4 days after electroporation in (**b**) and (**c**) (*n* = 3 pregnant females in each condition). Error bars represent SEM. **: *p* < 0.01, ***: *p* < 0.001, ****: *p* < 0.0001. One-way ANOVA; post hoc: Bonferroni test (color figure online)

Previously, loss of NDEL1 function was shown to lead to defects in neuronal migration in the developing mouse cortex [54]. To examine the effects of NDEL1 dysfunction on cortical development, we used short hairpin RNA (shRNA) to knock down *Ndel1* expression. The knockdown efficiency of four shRNAs targeting the coding region (sh*Ndel1*#13 and #14) or 3’ UTR (sh*Ndel1*#16 and #69) of *Ndel1* was tested in cultured primary neurons; sh*Ndel1*#16 showed the most significant reduction in NDEL1 protein expression **(**Fig. 3a**)**.

To confirm the effects of *Ndel1* knockdown on cortical development, we introduced these sh*Ndel1* constructs and green fluorescent protein (GFP) into the cerebral cortex of mouse embryos at E14.5 by in utero electroporation (IUE). Four days later, GFP + cells were examined in mouse brain sections by fluorescent confocal microscopy **(**Fig. 3b**; Supplementary Fig. 3a)**. In brains electroporated with control shRNA (shCtrl), most GFP + cells were distributed in the CP (77.2 ± 6.1%); few cells remained in the VZ/SVZ (10.3 ± 2.6%) and lower IZ (12.5 ± 3.6%, *n* = 3 pregnant mice in each condition). In contrast, brains electroporated with each *Ndel1* shRNA resulted in altered neuronal distribution with varying severity compared with control brains. In the most severe case, sh*Ndel1*#16 (referred to as sh*Ndel1* hereafter) caused a dramatic decrease in GFP + cells in the CP (26.0 ± 11.5%); most cells accumulated in the IZ (36.5 ± 0.6%) and SVZ (37.6 ± 4.8, *n* = 3 animals) **(**Fig. 3b, d**)**. This phenotype suggested defects in neuronal migration during cortical development [37, 67]. Interestingly, the severity of the altered cell distribution correlated well with the knockdown efficiency of the respective shRNA **(Supplementary Fig. 3a, b)**.

To test the potential functional impairment of the p.R105P variant on cortical development, we co-electroporated wild-type (WT) *Ndel1* or the p.R105P variant along with sh*Ndel1* into E14.5 embryos **(**Fig. 3b, d**; Supplementary Fig. 3c)**. We found that expression of *Ndel1* WT rescued the altered cell distribution caused by sh*Ndel1* (VZ/SVZ: 6.4 ± 2.0%; IZ: 16.3 ± 3.1%; CP: 77.3 ± 3.6%, *n* = 3 animals). In contrast, expression of p.R105P did not rescue the altered cell distribution and resulted in a more dramatic accumulation of cells in the IZ (SVZ: 13.0 ± 4.5%; IZ: 86.3 ± 4.7%; CP: 0.9 ± 0.2%, *n* = 3 animals), indicating an even stronger negative effect of p.R105P on neuronal migration **(**Fig. 3b, d**)**.

### NDEL1 p.R105P disrupted neuronal migration but not differentiation

Based on the above observation, we speculated that expression of the p.R105P variant alone could cause defects in neuronal migration. Therefore, we electroporated *Ndel1* WT, the p.R105P variant, or the empty vector along with GFP into E14.5 mouse embryos by IUE and examined cell distribution in brain slices after 4 days **(**Fig. 3c**)**. In control brains electroporated with the empty vector, most GFP + cells migrated to the CP (VZ/SVZ: 4.7 ± 2.1%, IZ: 11.6 ± 4.3%, CP: 83.6 ± 6.4%, *n* = 3 animals). Similar cell distribution patterns were also observed in brains electroporated with *Ndel1* WT, with most cells distributed in the CP (VZ/SVZ: 11.0 ± 4.8%, IZ: 18.9 ± 3.3%, CP: 70.0 ± 8.2%, *n* = 3 animals). Surprisingly, expression of p.R105P resulted in an accumulation of cells in the lower IZ and VZ/SVZ, with an almost complete loss of cells in the CP (VZ/SVZ: 24.4 ± 2.4%, IZ: 73.2 ± 2.6%, CP: 2.1 ± 0.7%, *n* = 3 animals) **(**Fig. 3c, d**)**.

Given the existence of two benign variants, p.Arg105Gln (p.R105Q) and p.Arg105Trp (p.R105W), at the same residue, we further investigated whether these variants could lead to migration defects similar to p.R105P. Thus, we examined the cell distribution of neurons electroporated with these benign variants in embryonic mouse brains. Notably, we observed relatively normal distributions of neurons expressing p.R105Q (VZ/SVZ: 8.7 ± 1.7%, IZ: 18.6 ± 4.1%, CP: 72.7 ± 5.7%, n = 5 animals) and p.R105W (VZ/SVZ: 14.2 ± 2.3%, IZ: 18.2 ± 2.9%, CP: 67.6 ± 3.9%, n = 5 animals), suggesting that these variants did not impair neuronal migration **(**Fig. 3c, d**)**. Furthermore, to test whether the cell accumulation in the IZ and VZ/SVZ is a neuronal effect, we expressed *Ndel1* WT and p.R105P specifically in post-mitotic neurons by IUE of *NeuroD1* promoter-driven plasmids (Fig. 3c). We found that expression of NDEL1 p.R105P in post-mitotic neurons severely disrupted neuronal migration (VZ/SVZ: 39.0 ± 0.9%, IZ: 59.5 ± 2.0%, CP: 1.5 ± 1.4%, n = 3 animals) **(**Fig. 3c, d**)**. These results suggested that post-mitotic p.R105P expression by itself impairs neuronal migration during cortical development.

**Fig. 4:**
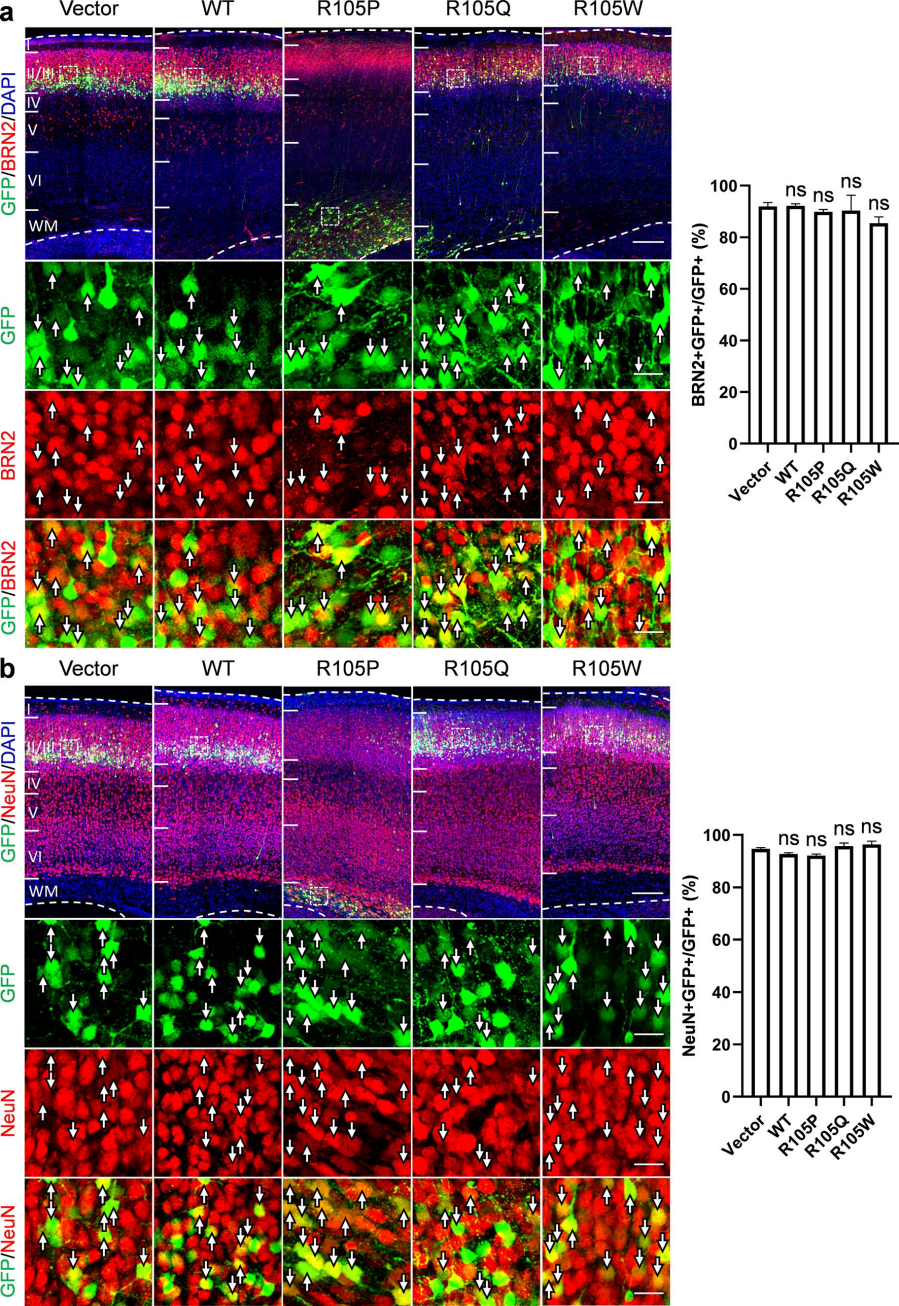
Expression of neuronal markers in cells expressing NDEL1 WT or p.R105P at P7. **a** Coronal sections at P7 from mouse brains electroporated with *Ndel1* WT, p.R105Q, p.R105W, p.R105P, or the empty vector (green) at E14.5 were stained with layer II/III marker, BRN2 (red). Most of the cells electroporated with *Ndel1* WT, p.R105Q, p.R105W, and p.R105P were BRN2 + , similar to the control cells electroporated with the empty vector, even though p.R105P-expressing cells were arrested in the WM. **b** Similarly, in brains electroporated with *Ndel1* WT, p.R105Q, p.R105W, p.R105P, and the empty vector, most of the electroporated cells were NeuN + (red). The regions within the white boxes were magnified. Bars = 200 μm in the wide field images and 20 μm in high magnification images. Bar graphs show the percentage of BRN2 + /GFP + and NeuN + /GFP + cells in the electroporated brain slices (*n* = 3 mice from 3 independent pregnancies). Error bars represent SEM. One-way ANOVA; post hoc: Bonferroni test. ns: not significant (color figure online)

Previous studies have shown that post-mitotic cells generated by RGCs at E14.5 mostly differentiate into pyramidal neurons of layer II/III in the cerebral cortex [37]. To further investigate whether p.R105P expression in the brain could also affect neuronal differentiation at later developmental stages, we stained brain slices at postnatal day 7 (P7) with the neuronal marker NeuN and the upper cortical layer marker BRN2 after IUE at E14.5 **(**Fig. 4**)**. In brains electroporated with the control empty vector, most GFP + cells were observed in layer II/III, and these cells were also NeuN + (94.7 ± 0.4%) and BRN2 + (91.9 ± 1.6%, *n* = 3 animals). Similarly, in brains electroporated with *Ndel1* WT and its benign variants, most GFP + cells also expressed NeuN (WT: 92.7 ± 0.5%, p.R105Q: 95.7 ± 1.2%, p.R105W: 96.3 ± 1.3%, *n* = 3 animals in all groups) and BRN2 (WT: 92.2 ± 0.8%, p.R105Q: 90.3 ± 6.0%, p.R105W: 85.4 ± 2.5%, *n* = 3 animals in all groups). Interestingly, although p.R105P-expressing cells did not migrate to the CP, the majority of these cells were still positive for NeuN (92.1 ± 0.6%) and BRN2 (89.9 ± 0.9%, *n* = 3 animals). These results suggested that expression of p.R105P can disrupt neuronal migration with minimal effects on neuronal differentiation.

### NDEL1 p.R105P increased the length of the leading process and the N–C distance

**Fig. 5:**
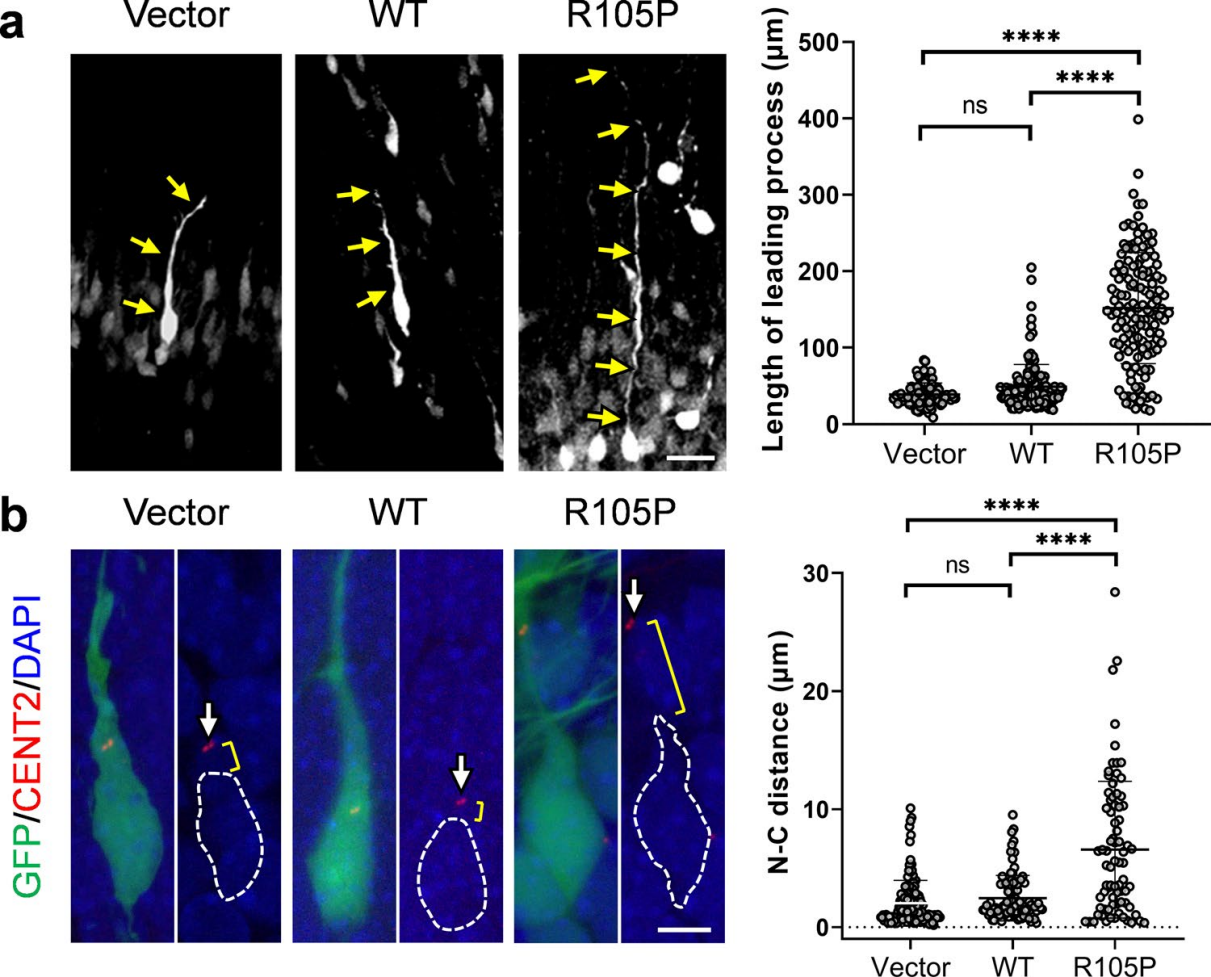
NDEL1 p.R105P increased the length of the leading process and the nucleus–centrosome distance (N–C distance). **a** Representative images of GFP + neurons in E18.5 mouse brain electroporated with empty vector, *Ndel1* WT or p.R105P cDNA along with GFP (green) at E14.5. Arrows represent the leading process from the cell body. Bars = 20 μm. Cells expressing p.R105P extended a very long leading process compared to those cells electroporated with the empty vector or *Ndel1* WT. The spot graph shows the length of the leading process in cells electroporated with the empty vector (*n* = 92 cells, acquired from 3 mice), *Ndel1* WT (*n* = 138 cells, acquired from 3 mice), or p.R105P (*n* = 140 cells, acquired from 3 mice). Error bars represent SEM. ****: *p* < 0.0001. Kruskal–Wallis test; post hoc: Dunn’s test. ns: not significant. **b** Representative images of migrating neurons electroporated with *Ndel1* WT or p.R105P cDNA along with GFP (green) and CentrinII-DsRed (red). The brains were electroporated at E14.5 and harvested at E17.5. The centrosomes (arrowhead) in most cells expressing empty vector and *Ndel1* WT were found in the perinuclear area, while the centrosomes in cells expressing p.R105P were often found further along the leading process at a longer distance from the nucleus (dashed lines). Spot graph shows the N–C distance in cells electroporated with empty vector (*n* = 130 cells, acquired from 3 mice), *Ndel1* WT (*n* = 91 cells, acquired from 3 mice), or p.R105P (*n* = 77 cells, acquired from 3 mice). Error bars represent SEM. ****: *p* < 0.0001. Kruskal–Wallis test; post-hoc: Dunn’s test. ns: not significant (color figure online)

As a member of the cytoplasmic dynein complex, the function of NDEL1 may be involved in the extension of the leading process and translocation of the centrosome, which could be evaluated through cell morphology [54]. Interestingly, most cells arrested in the IZ of the brains electroporated with p.R105P appeared to exhibit bipolar morphology. Remarkably, we observed dramatically long leading processes extending from the cell body toward the CP **(**Fig. 5a**)**. The length of the leading process in cells expressing p.R105P was significantly longer than those in cells electroporated with the empty vector or WT *Ndel1* (vector: 38.8 ± 1.6 μm, WT: 48.9 ± 2.5 μm, p.R105P: 151.9 ± 6.1 μm).

After the leading process extension, the centrosome migrates into the leading process prior to nuclear translocation [6, 53, 58, 66, 67]. To further test whether our p.R105P variant could affect this process, we examined the distance between the nucleus and the centrosome (N–C distance). *Ndel1* WT, p.R105P, or the empty vector was electroporated along with the centrosome marker CentrinII-dsRed and GFP at E14.5. Brain sections were harvested at E17.5, and the centrosomes and nuclei were imaged by fluorescent confocal microscopy **(**Fig. 5b**)**. We found that the N–C distance was significantly increased in cells expressing p.R105P compared with those electroporated with *Ndel1* WT or the empty vector (vector: 2.0 ± 0.2 μm, WT: 2.5 ± 0.2 μm, p.R105P: 6.6 ± 0.7 μm), suggesting defects in N–C coupling during neuronal migration.

### NDEL1 p.R105P disrupted interaction with LIS1 and centrosomal localization

**Fig. 6:**
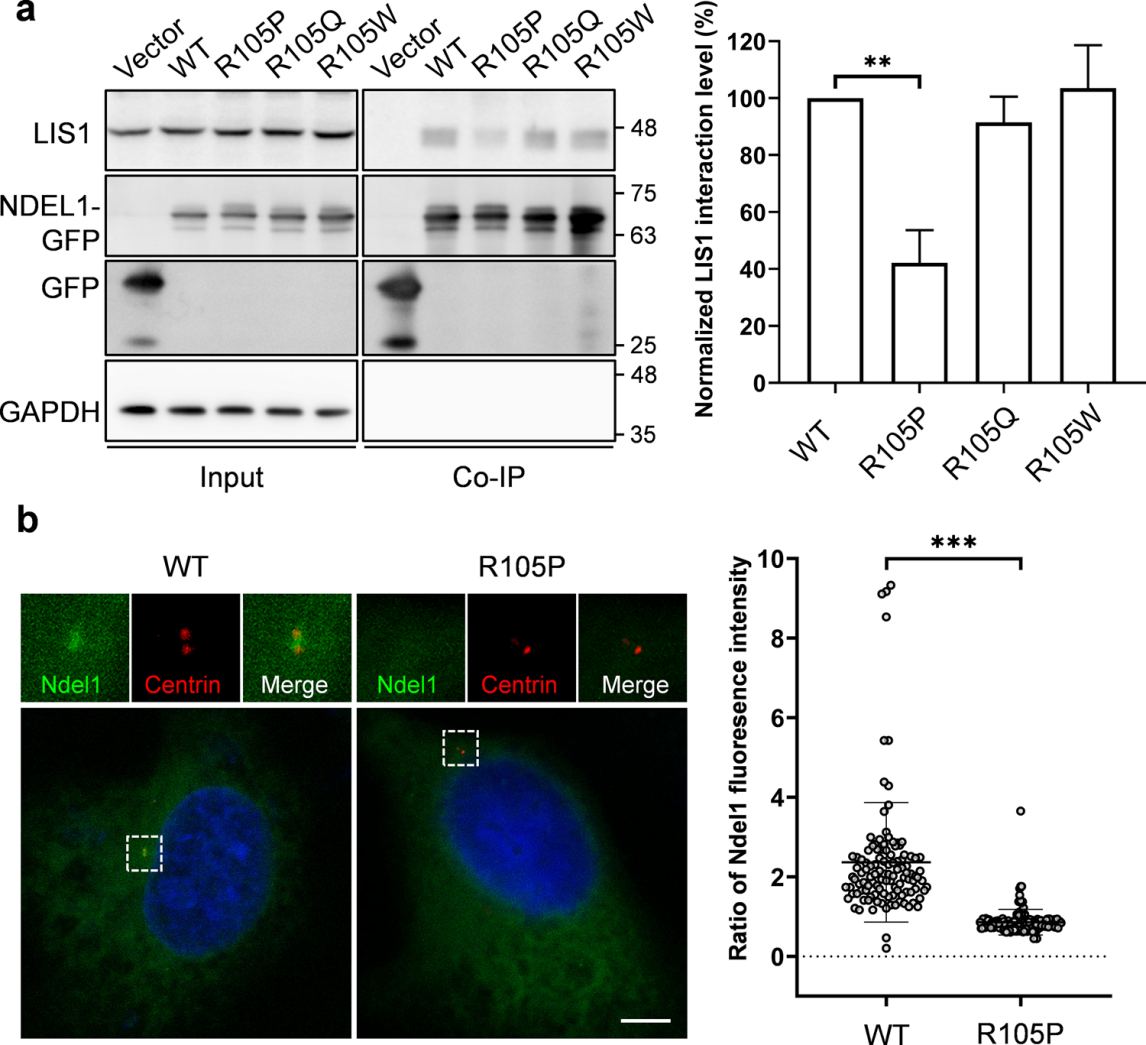
The p.R105P variant disrupted NDEL1 interaction with LIS1 and decreased the localization at the centrosome. **a** Immunoprecipitation of NDEL1 WT and p.R105P to LIS1. Lysates from HEK293T cells transfected with *Ndel1* WT, p.R105Q, p.R105W, or p.R105P for 24 h were immunoprecipitated by GFP-antibody. Western blots show that LIS1 co-precipitated with NDEL1 WT, p.R105Q, and p.R105W, but not the p.R105P variant. The bar graph shows co-precipitated LIS1 with p.R105Q (*n* = 5), p.R105W (*n* = 5), and p.R105P (*n* = 8) relative to NDEL1 WT (*n* = 8). Error bars represent SEM. **: *p* < 0.01, Kruskal–Wallis test; post hoc: Dunn’s test. Data of each repeat in each group were acquired from independent batches of experiments. **b** Centrosomal localization of NDEL1 WT and the p.R105P variant. U2OS cells were transfected with pEGFP-mNDEL1(WT) or pEGFP-mNDEL1(R105P) (green) for 24 h and stained with the centrosome marker CentrinII (red). The regions within the box were magnified. Bar = 5 μm. The spot graph shows the relative intensity of NDEL1 fluorescence at the centrosome to the cytosol. There was a dramatic decrease in the localization of p.R105P (*n* = 138 cells) at the centrosome compared to WT (*n* = 114 cells). Error bars represent SEM. ****: *p* < 0.0001. Mann–Whitney test. Data from each group were acquired from three independent batches of experiments (color figure online)

As a regulator of cytoplasmic dynein, NDEL1 plays a critical role in dynein function [35, 59]. Mutations in the NDEL1-interacting protein LIS1 also resulted in lissencephaly, suggesting a common role in cortical development [26, 42, 67]. Because the mutation site of NDEL1 p.R105P was at the binding site to LIS1, we performed co-immunoprecipitation (co-IP) to investigate whether p.R105P disrupted protein interactions in HEK293T cells transfected with constructs expressing EGFP-tagged NDEL1 (pEGFP-NDEL1) or the p.R105P (pEGFP-R105P), p.R105Q (pEGFP-R105Q), and p.R105W (pEGFP-R105W) variants. We found that the interaction between NDEL1 and LIS1 was largely abolished by the p.R105P variant, whereas the p.R105Q and p.R105W variants did not exhibit such an effect **(**Fig. 6a**)**. This suggested that p.R105P drastically reduces the interaction between NDEL1 and the LIS1-dynein complex, which may underlie the impairment of neuronal migration during cortical development.

Previously, NDEL1 was reported to localize to the centrosome [23, 45]. Therefore, we next examined whether the p.R105P variant could affect the subcellular localization of NDEL1 to the centrosome. pEGFP-NDEL1 or pEGFP-R105P were transfected into cultured U2OS cells for 24 h. Consistent with previous studies, NDEL1 WT specifically concentrated at the centrosome, with some signal spreading in the cytosol **(**Fig. 6b**)**. In contrast, the NDEL1 p.R105P variant distributed mainly in the cytosol without localizing to the centrosome** (**relative fluorescence intensity: WT: 2.6 ± 0.2, *n* = 66 cells; p.R105P: 0.9 ± 0.04, *n* = 84 cells). This result suggested that p.R105P decreases the localization of NDEL1 to the centrosome.

## Discussion

**Fig. 7:**
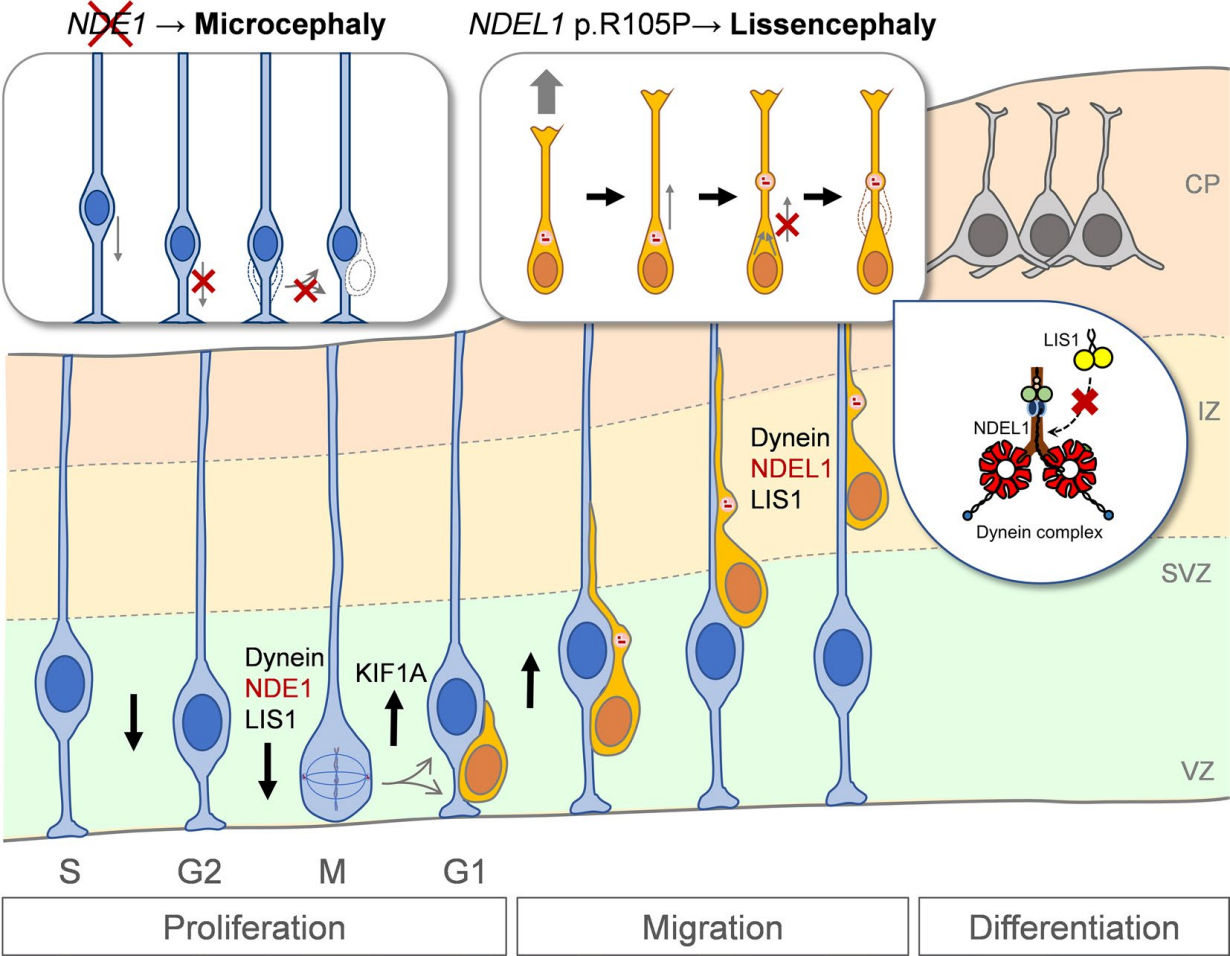
Schematic diagram depicting the distinct roles of NDE1 and NDEL1 in cortical development and the cellular and molecular mechanisms of NDEL1 p.R105P variant in causing lissencephaly. During cortical development, the nucleus of RGCs undergoes cell-cycle-dependent INM during proliferation to self-renew and produce neurons. Cytoplasmic dynein, supported by NDE1 and LIS1, facilitates downward movement, while KIF1A is responsible for upward movement. Dysfunction of NDE1 blocks downward movement and subsequent proliferation; its mutations lead to microcephaly in humans. Post-mitotic neurons migrate along the radial fibers of RGCs to populate the CP, where they become pyramidal neurons. During the migration process, NDEL1 interacts with both cytoplasmic dynein and LIS1 to regulate dynein motor activities, enabling nucleokinesis during neuronal migration. The p.R105P variant disrupts the interaction of NDEL1 with LIS1, which impairs the function of the cytoplasmic dynein complex. This defect impedes N–C coupling, blocks nucleokinesis, and impairs neuronal migration during cortical development. These defects, in turn, lead to the pathogenesis of human lissencephaly

In this study, we identified two patients with pachygyria, with or without SBH, both carrying the same postzygotic mosaic pathogenic *NDEL1* variant, p.R105P **(**Fig. 1**)**. scRNA-seq and spatial transcriptomic analysis unveiled a complementary expression pattern of *Nde1*/*NDE1* and *Ndel1*/*NDEL1* in the developing cortex **(**Fig. 2**)**. Subsequently, we showed that expression of p.R105P failed to rescue the migration defect caused by *Ndel1* knockdown. Interestingly, the expression of p.R105P alone strongly disrupted neuronal migration but had no effect on neuronal differentiation **(**Fig. 3, 4**)**. Cells arrested in the IZ exhibited a longer leading process and increased N–C distance **(**Fig. 5**)**. Mechanistically, the p.R105P variant reduced the interaction of NDEL1 with LIS1 and its localization to the centrosome in cells **(**Fig. 6**)**. These defects may impair N–C coupling and nucleokinesis during neuronal migration, potentially contributing to the pathogenesis of lissencephaly **(**Fig. 7**)**.

Clinically, both patients exhibited psychomotor delay, with predominant language impairment and drug-resistant epilepsy. Their MRI revealed a similar pattern of neuronal migration disorder, characterized by pachygyria and mild thickening of the corpus callosum. In the second case, posterior migration abnormalities manifested as double cortex, with normal subcortical U-fibers, while in the anterior regions, the migration disorder presented as diffuse pachygyria. The clinical and neuroimaging presentation of the first case was more severe than that of the second, potentially corresponding to the degree of mutation burden. Somatic mosaicism of other lissencephaly genes, such as *LIS1* and *DCX*, has previously been reported to cause SBH in addition to pachygyria [50, 55, 71]. It has been suggested that the frequency of somatic variants is associated with the severity of clinical symptoms [1, 55]. Migrating neurons with pathogenic variants are more likely to arrest on the path to the CP, hence separating from those with WT alleles. In contrast, both posterior predominant SBH and pachygyria have been independently observed in patients resulting from apparently germline *CEP85L* variants [14, 30, 33, 43, 71]. It is possible that other genetic or environmental factors affecting neuronal migration also play a role in modulating the severity of the disease.

Interestingly, as seen in Case 1, a posterior predominant gradient has been observed in lissencephaly patients with pathogenic *LIS1* and *CEP85L* variants. In contrast, Case 2 presented with anterior pachygyria and posterior SBH, a pattern observed in patients with pathogenic *DCX* variants. It remains unclear why different brain areas are preferentially affected by different genes or even in different individuals, but previous evidence suggests that it may be caused by the differential expression of these genes during brain development [30]. Taken together, the complex interaction between somatic mutation burden and preferential gene expression pattern likely determines the location and severity of cortical malformation.

Although NDE1 and NDEL1 are very similar in their a.a. sequence (56% identical and 69% similar in humans) [4, 56], they show different expression patterns during development. Previously, *NDE1* was found to be highly expressed for a few months after fertilization and then downregulated in most tissues [11], whereas the expression of *NDEL1* was found to be relatively constant [9, 11]. Here, using scRNA-seq and spatial transcriptomic analysis, we obtained the first expression profiles of *Ndel1*/*NDE1* and *Nde1*/*NDEL1* in the developing cerebral cortex at the single-cell level with spatial resolution. We found that *Nde1*/*NDE1* was mainly expressed in RGCs in the VZ during the G2/M phase, and *Ndel1*/*NDEL1* was expressed in differentiated neurons in the IZ and CP, consistent with previous studies using immunofluorescence staining [20, 45, 48]. This finding is consistent with the gene expression studies mentioned above and suggests distinct roles of NDE1 and NDEL1 during cortical development. Clinically, our patients exhibit a prominent neuronal migration deficit characterized by lissencephaly and SBH without other associated dysmorphology of the cerebellum, brain stem, or basal ganglion. Unlike patients with *NDE1* mutations, our patients did not exhibit microcephaly, suggesting that the p.R105P variant does not have a major impact on neurogenesis. The presence of microcephaly could be a distinguishable clinical phenotype of the two closely related genes.

NDE1 and NDEL1 are involved in the positioning of the nucleus in many developmental processes [10, 19, 34, 41, 49, 75, 76]. It has been reported that NDE1, together with cytoplasmic dynein, dynactin, and LIS1, facilitates the downward nuclear movement of RGCs during INM [16, 18, 65]. Here, we found that NDE1 is specifically expressed at the G2/M phase when the downward nuclear movement occurs. This finding suggests a model in which dynein is activated by the expression of NDE1 and is engaged for downward nuclear movement during the G2 phase. After mitosis, the expression of NDE1 is eliminated, thus inactivating dynein from nuclear movements. KIF1A is next engaged with the nucleus and facilitates upward nuclear movement during the G1 phase [15, 65]. Intriguingly, the knockdown of *Ndel1* did not affect INM, ruling out the possible role of NDEL1 in this process [18]. In contrast, the knockdown of *Ndel1* disrupted the migration of neurons and increased the N–C distance in these cells, suggesting its role solely in nucleokinesis during neuronal migration [54, 79]. Although the knockdown of *Nde1* also arrested cells in the VZ/SVZ of the developing mouse cerebral cortex [18, 54], this may be due to its effects on RGC proliferation rather than neuronal migration. Remarkably, exogenous NDE1 or NDEL1 expression could cross-rescue the defects from knockdown of each other, suggesting their overlapping functions in nuclear migration [18]. Our results, therefore, suggest that the different phenotypes of NDE1 and NDEL1 dysfunction in humans and mice are due to differences in expression rather than their molecular functions.

During neuronal migration, microtubules form a cage-like structure that surrounds the nucleus and facilitates nucleokinesis [54, 62, 67]. Knockdown of the dynein-associated proteins NDEL1 and LIS1 impairs nucleokinesis [54, 67]. Previously, NDEL1 was shown to be involved in microtubule network organization [23, 81]. Therefore, it is likely that the pathogenic *NDEL1* variant impairs nucleokinesis via interaction with the cytoplasmic dynein complex, which is supported by the increased N–C distance. In addition, NDEL1 and LIS1 have been shown to regulate centrosome orientation in migrating neurons, affecting their direction of migration [79]. These NDEL1 functions ultimately regulate neuronal migration during cortical development.

Neuronal migration in the developing cortex is a sequential process that spans from embryonic to postnatal stages. Utilizing IUE, the level of migration delay can be evaluated from E18.5 to P7 after electroporation [13, 28, 37, 67, 70, 71]. If neurons fail to reach their final cortical layer by P7, these cells are considered arrested. Here, our results showed that the expression of the pathogenic p.R105P variant alone led to migration delay at E18.5. Subsequently, these cells differentiated into BRN2 + NeuN + cortical neurons and accumulated in the IZ at P7. This data suggests that the p.R105P variant may result in the arrest of BRN2 + migrating neurons within deeper cortical layers or potentially induce the premature differentiation of BRN2 + neurons, followed by a disruption in their migrating processes.

NDEL1 has two dynein-binding domains, one near the N-terminus that interacts with the dynein intermediate chain [80] and the other in the α-helix within the C-terminus that binds to the dynein heavy chain [52]. The LIS1-binding domain is located in the N-terminal coiled-coil domain and is distinct from the dynein-binding domains [78]. However, NDEL1 can fold to bring the C-terminal dynein-binding domain into proximity with the N-terminal dynein- and LIS1-binding domains [57]. In our p.R105P variant, the substitution of arginine with proline has the potential to introduce a kink in the coiled-coil domain, thereby affecting its binding ability to the LIS1 protein. Indeed, we found that the NDEL1 p.R105P variant dramatically reduces its binding ability to LIS1, supporting this notion. Interestingly, the benign variants p.R105Q and p.R105W at the same residue may not exert a similar effect on the coiled-coil domain, suggesting that p.R105P has a distinct impact on the molecular structure and functions of NDEL1.

How do defects in the NDEL1-LIS1 interaction affect neuronal migration at the molecular level? Previously, a “clutch” model of dynein motion under high loads was proposed [8, 29]. In this model, NDEL1 or NDE1 binds to the dynein intermediate and light chains with its C-terminal binding domain. When the coiled-coil domain recruits LIS1 along with the N-terminal dynein-binding domain, it positions LIS1 near the dynein motor domain. This process strengthens the interaction between cytoplasmic dynein and microtubules, enables the summation of multiple dynein forces, and reduces the speed of dynein motion. The “clutch” is necessary for the transport of high-load cargo [39, 40], such as the nucleus. Disrupted association of LIS1 and NDEL1 can, thus, lead to failure of nucleokinesis during neuronal migration.

In summary, we have identified the first two human lissencephaly patients caused by the de novo somatic variant p.R105P of *NDEL1*, a long-expected potential gene for cortical malformations. This mutant abolished the interaction of NDEL1 with LIS1, an important component of the cytoplasmic dynein complex. This, in turn, caused defects in N–C coupling and ultimately led to defects in neuronal migration. Moreover, the complementary expression patterns of *Nde1*/*NDE1* and *Ndel1*/*NDEL1* in the developing cerebral cortex also shed light on their distinct roles in cortical development and pathogenesis of human MCDs.

### Supplementary Information

Below is the link to the electronic supplementary material.

Supplementary file1 (PDF 1098 KB)

## Data Availability

All data and materials are contained within the paper and the files or available on reasonable request. The scRNA-seq data is available at 10.57770/IWASQL. The spatial gene transcriptome data is available at 10.57770/P3TK72.
