# Supplementary material for: Novel lissencephaly-associated NDEL1 variant reveals distinct roles of NDE1 and NDEL1 in nucleokinesis and human cortical malformations
Source: Acta Neuropathol. 2024 Jan 9;147(1):13. doi: 10.1007/s00401-023-02665-y (PMC10776482; doi:10.1007/s00401-023-02665-y)
Supplement: Supplementary file 1 — Supplementary file1 (PDF 1098 KB) [file 401_2023_2665_MOESM1_ESM.pdf]

Supplementary Figures

E13.5 mouse

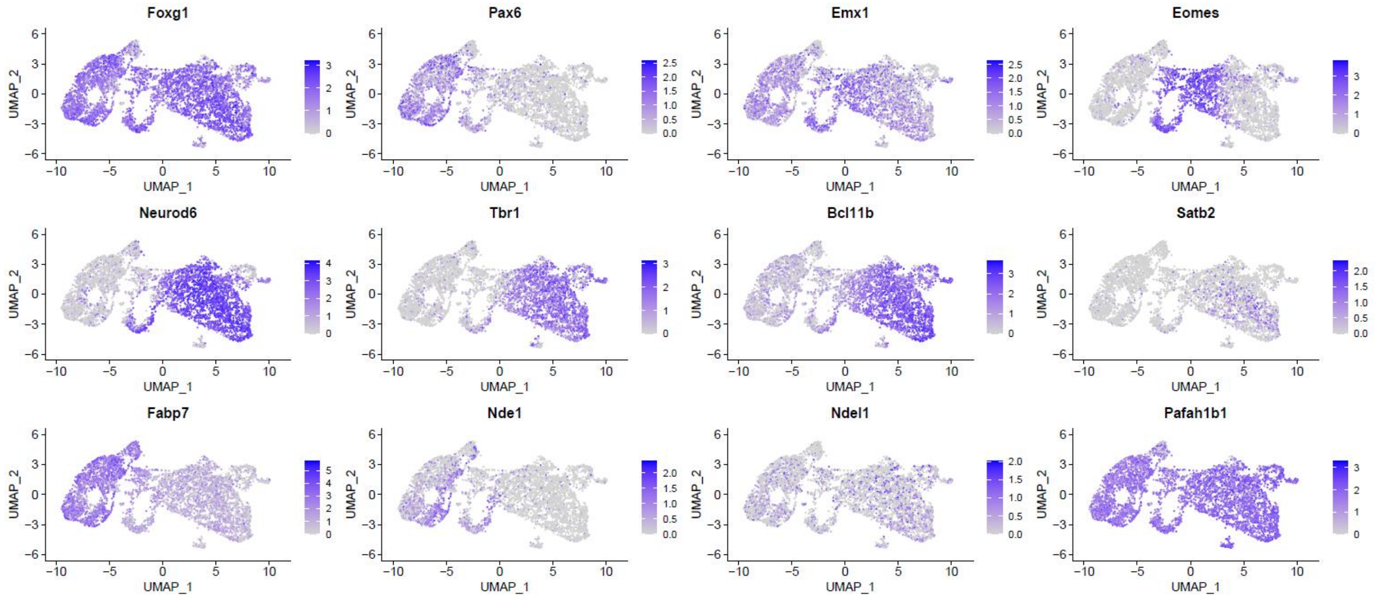

E15.5 mouse

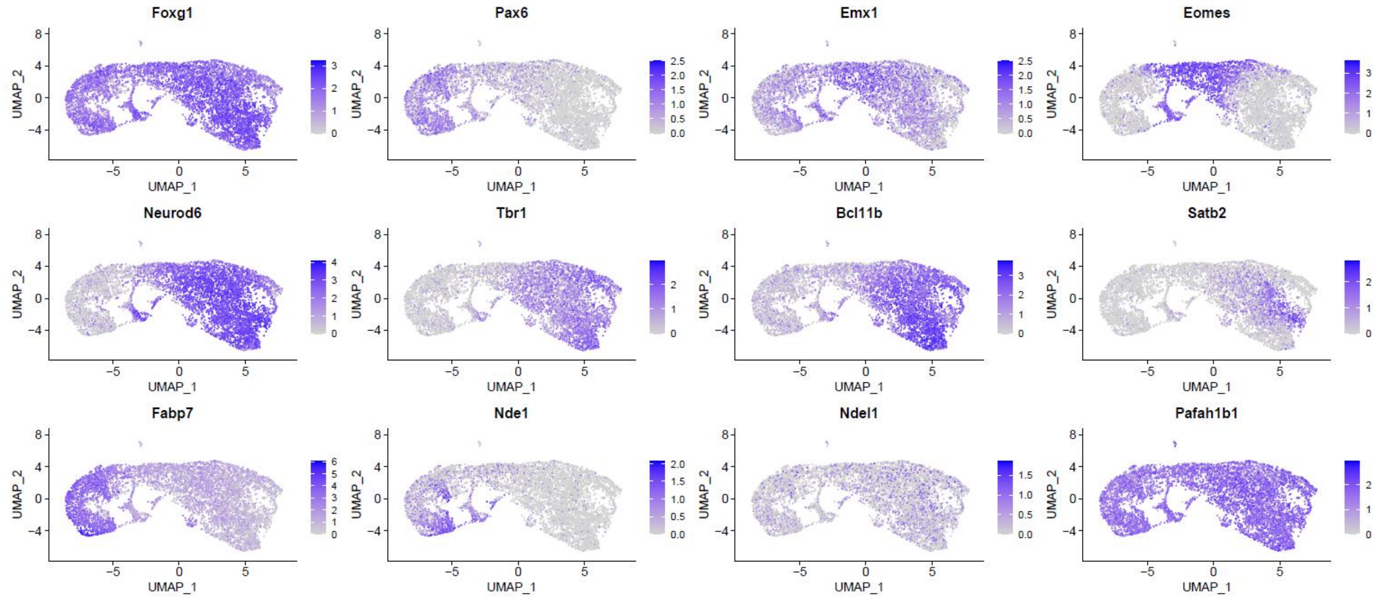

GW18 human

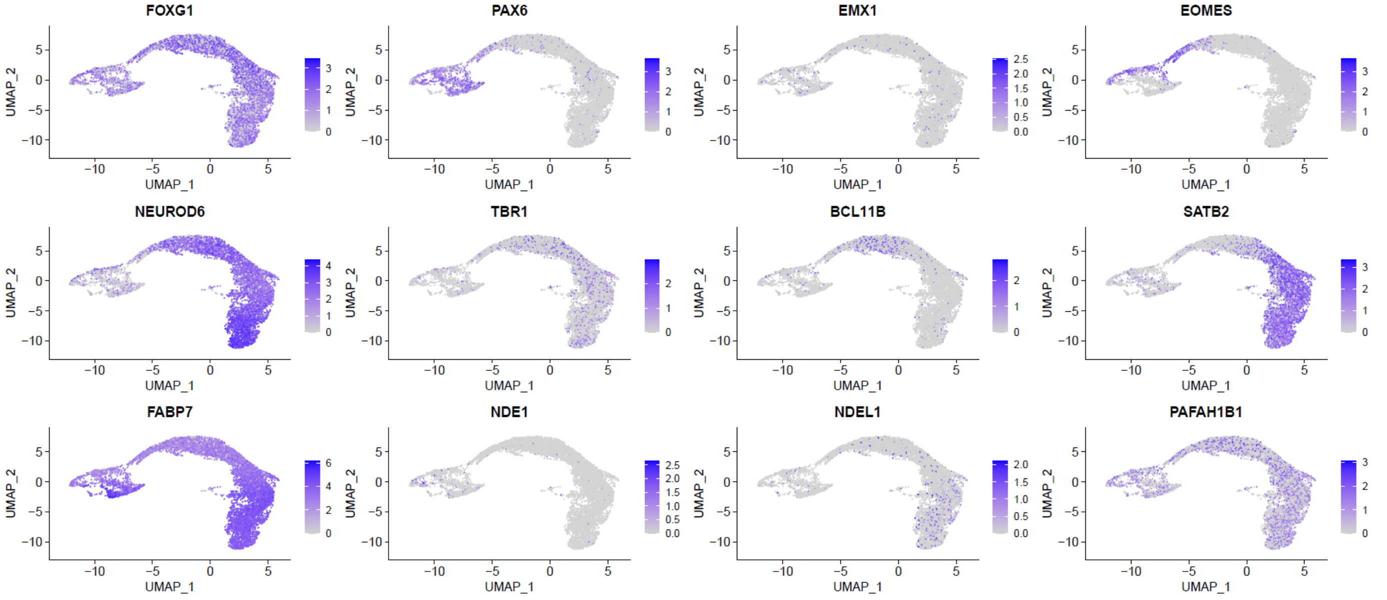

Supplementary Figure 1. Expression of the markers for different neuronal developmental states of E13.5, E15.5 mouse, and GW18 human cortices. The heat maps of UMI counts in each gene are mapped into the UMAP plot from scRNA-seq data. Expression of the marker genes for progenitors (*Pax6*/*PAX6* and *Fabp7*/*FABP7*), transient precursor stage (*Eomes*/*EOMES*), and postmitotic neurons (*Neurod6*/*NEUROD6*), as well as the cortex (*Tbr1*/*TBR1*, *Bcl11b*/*BCL11B*, and *Satb2*/*SATB2*) are shown. The color scale represents the log-normalization of the UMI count.

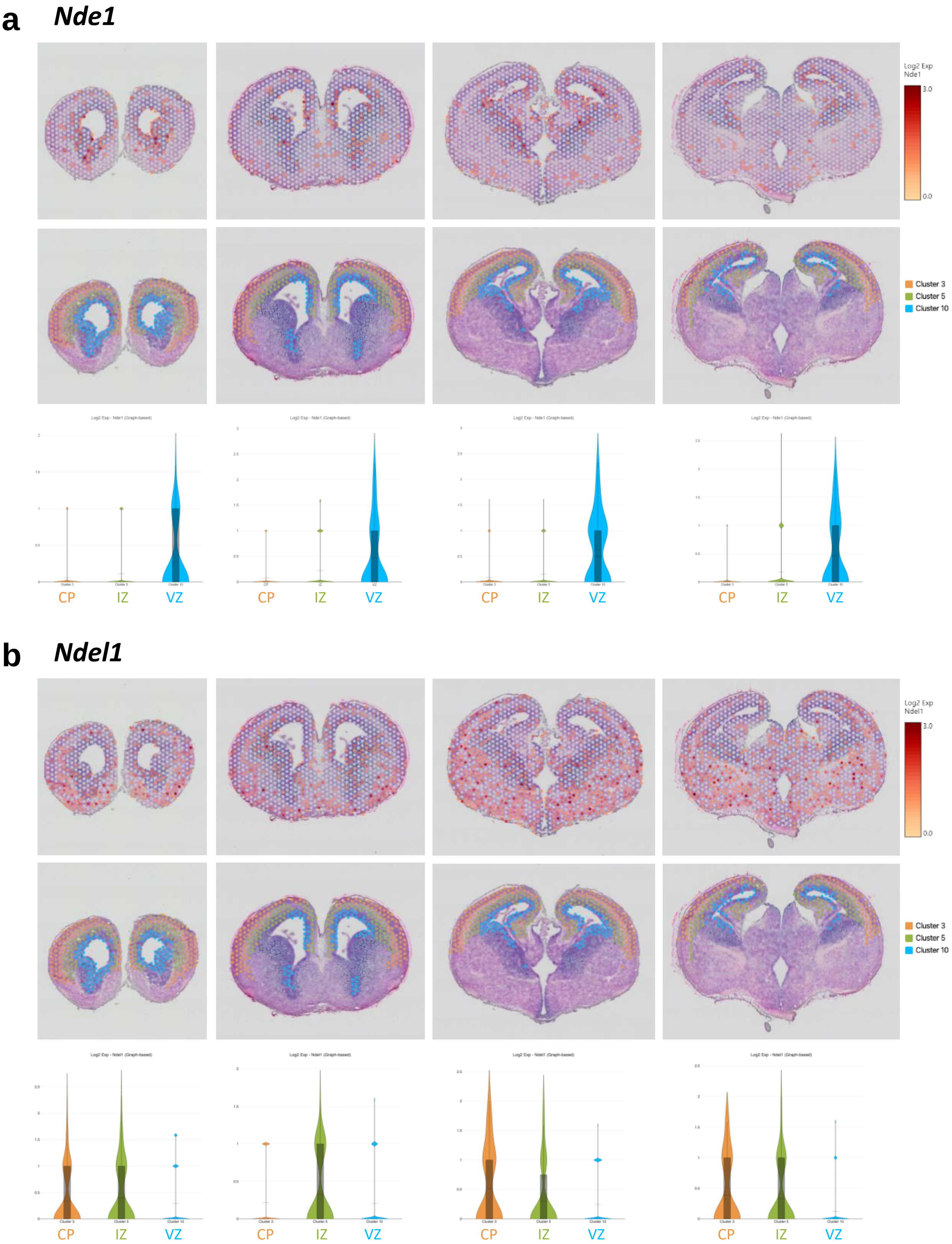

Supplementary Figure 2. Spatial gene expression profiles of *Nde1* and *Ndel1* in mouse telencephalon at E15.5. The spatial distribution and violin plot of the expression of *Nde1* (a) and *Ndel1* (b) in the dorsal telencephalon. While *Nde1* is strongly expressed in the VZ, *Ndel1* is mainly expressed in the IZ and CP. The color scale represents the log-normalization of the unique molecular identifier (UMI) count.

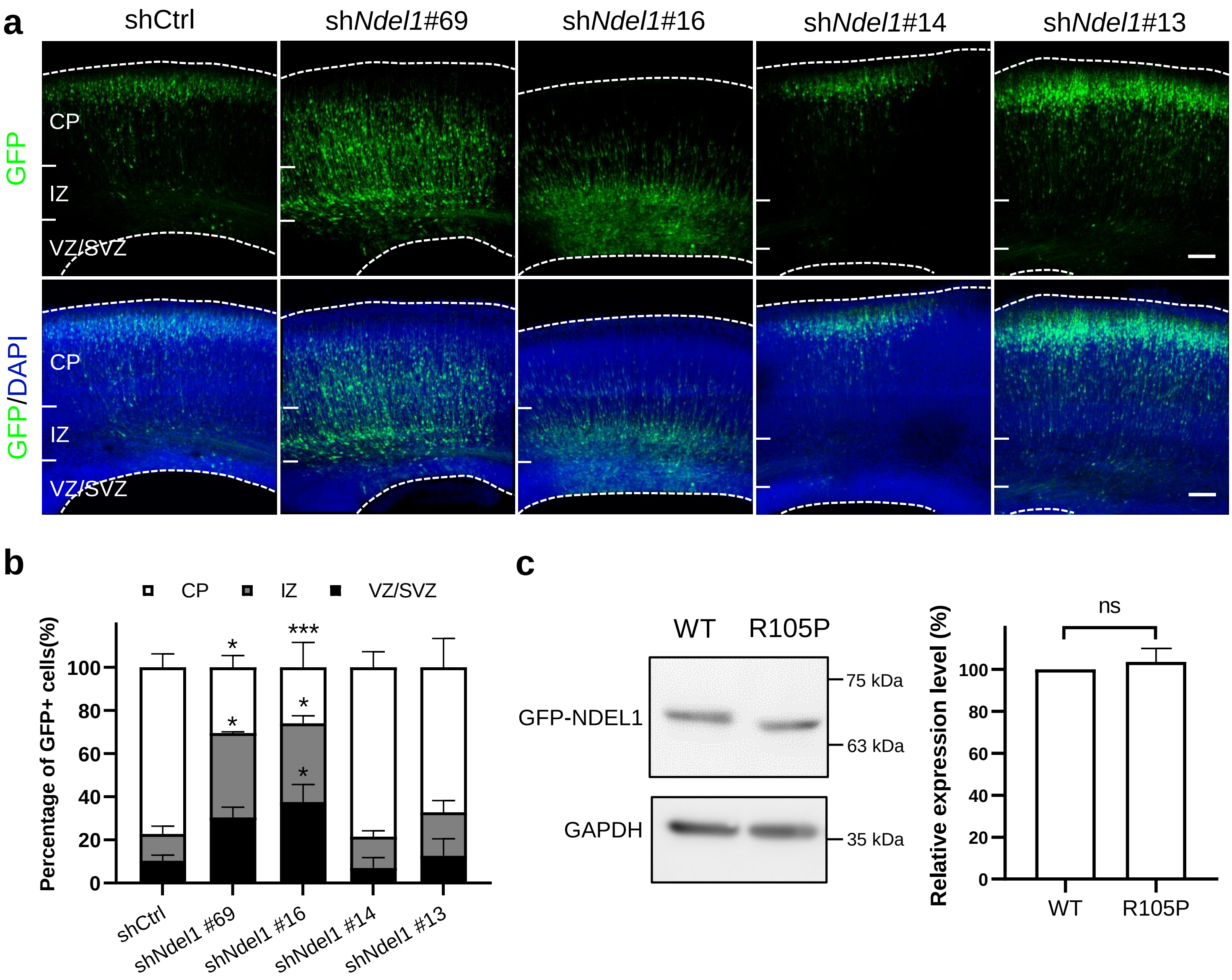

Supplementary Figure 3. Cell distribution in the developing cortex subjected to *Ndel1* shRNA. (a) Coronal sections of brains electroporated with shCtrl or sh*Ndel1* along with GFP (green) at E14.5 were collected 4 days after *in utero* electroporation. The severity of cell migration delay correlated well with the knockdown efficiency of respective shRNA, as shown in Figure 3a. Bar = 100 μm. (b) The bar graph shows the percentage of GFP+ cells in the CP, IZ, and VZ 4 days after electroporation (n = 3 pregnant mice in each condition). Error bars represent SEM. *: $p < 0.05$, ***: $p < 0.001$. One-way ANOVA; post-hoc: Bonferroni test. (c) Western blot analysis showing the expression of NDEL1 in HEK293T transfected with WT or p.R105P cDNA. The bar graph shows similar expression levels between WT and p.R105P variant. (n = 3 independent experiments). Error bars represent SEM. Student's *t*-test. ns: not significant.
